# Differential Effects of Transcranial Direct Current Stimulation (tDCS) Depending on Previous Musical Training

**DOI:** 10.3389/fpsyg.2018.01465

**Published:** 2018-09-10

**Authors:** Ana Sánchez-Kuhn, Cristian Pérez-Fernández, Margarita Moreno, Pilar Flores, Fernando Sánchez-Santed

**Affiliations:** ^1^Department of Psychology and CIAIMBITAL, CeiA3, University of Almería, Almería, Spain; ^2^Instituto de Neurorehabilitación Infantil InPaula, Almería, Spain

**Keywords:** transcranial direct current stimulation, motor cortex, sequential finger tapping task, musicians, individual differences

## Abstract

Previous studies have shown that transcranial direct current stimulation (tDCS) facilitates motor performance, but individual differences such as baseline performance seem to influence this effect. Accordingly, musicians offer an inter-individual differences model due to anatomical and functional variances displayed among the motor cortex regions. The aim of the present work was to study if the baseline motor skill predicts whether tDCS can enhance motor learning. For that objective, we administered anodal (*n* = 20) or sham (*n* = 20) tDCS on the right primary motor cortex region of 40 right-handed healthy participants, who were divided into four groups: musicians (tDCS/sham) and non-musicians (tDCS/sham). We measured the skill index (SI) presented in the sequential finger-tapping task (SEQTAP) at baseline, during three 20 min/2 mA stimulation sessions, and in follow-up tests after 20 min and 8 days. Depending on the normality of the data distribution, statistical differences were estimated by ANOVA and Bonferroni *post hoc* test or Kruskal–Wallis and U Mann–Whitney. Results showed that musicians scored higher in baseline performance than non-musicians. The non-musicians who received tDCS scored higher than the sham group in the first and second stimulation session. This effect was extended to the 20 min and 8 days follow-up test. In musicians, there was no effect of tDCS. The present method seems to be suitable for the achievement of positive and consolidated tDCS effects on motor learning in inexperienced participants, but not in musicians. These data may have an implication for the rehabilitation of motor impairments, contributing to more individualized stimulation protocols.

## Introduction

Transcranial direct current stimulation (tDCS) is a non-invasive neuromodulation technique that is garnering increasing interest as an innovative neurorehabilitation tool (Dubljeviæ et al., 2014; De Ridder et al., 2017). tDCS has shown to change the excitability of the underlying neurons of the stimulated cortical area (Nitsche and Paulus, 2000), while being safe and relatively easy to use (Bikson et al., 2016). There is an increasing body of evidence from tDCS studies reporting positive effects on human motor function (Russo et al., 2017; Sánchez-Kuhn et al., 2017). tDCS has contributed to the motor rehabilitation of motor deficits derived from stroke (Boggio et al., 2007; Russo et al., 2017), dysphagia (Kumar et al., 2011), and Parkinson’s disease (Fregni et al., 2006), and its applicability has been extended to cerebral palsy in children (Almeida et al., 2014; Aree-Uea et al., 2014). In healthy participants, it has been shown to enhance motor function in the upper limbs (Reis and Fritsch, 2011; Sriraman et al., 2014; Karok et al., 2017) and the lower limbs (van Asseldonk and Boonstra, 2016). Previous studies have confirmed that tDCS might afford the most substantial benefits when combined with motor training (Reis and Fritsch, 2011; Page et al., 2015; Pérez-Fernández et al., 2016).

Transcranial direct current stimulation is administered by a portable device that contains a 9-volt battery connected to two electrodes: the anode and the cathode. A constant mild electrical current flows between both electrodes, entering through the scalp and changing the excitability of the underlying neurons. This process generates depolarization or polarization of the neuronal membrane, depending on the anodal (a-tDCS) or cathodal (c-tDCS) nature of the electrode, respectively, although these effects can be reversed depending on the intensity and the duration of the stimulation (Nitsche et al., 2008). The administration of tDCS has been shown to produce sustainable changes in the amplitude of the motor evoked potentials of the cortical stimulated area that last for up to 90 min after stimulation ends (Nitsche and Paulus, 2001). Functional near-infrared spectroscopy (Merzagora et al., 2010; Takai et al., 2016) and positron emission tomography (Lang et al., 2005) have shown tDCS to increase cerebral blood flow and oxyhaemoglobin concentrations. Moreover, functional magnetic resonance imaging (fMRI) has shown activation of the primary motor cortex (M1) after stimulation by a-tDCS (Kwon et al., 2008), confirming its cortical excitatory effects.

The excitatory effects of tDCS have been attributed to an important reduction in GABA activity and a *N*-methyl-D-aspartate receptor (NMDAr)-mediated augmentation of synaptic strength via an increase in intracellular Ca^2+^ levels (Liebetanz et al., 2002). Moreover, the alteration of the glutamatergic system could ultimately lead to the release of brain-derived neurotrophic factor (BDNF) (Clarkson et al., 2010). In fact, treatment with tDCS has been shown to change BDNF levels (Filho et al., 2016), promoting BDNF-dependent synaptic plasticity (Fritsch et al., 2010), which might be key in explaining the long lasting effects of tDCS. Consequently, tDCS has shown to produce greater motor learning, compared to a sham condition, with a maintenance of these effects for up to 3 months, highlighting this technique as a promising neurorehabilitation tool (Reis et al., 2009). However, literature also shows a large amount of variability among the corticospinal excitability reactions of stimulated participants (Wiethoff et al., 2014). A recent meta-analysis concluded that the application of tDCS enhances motor skills but with rather low effect sizes (Hashemirad et al., 2016). The literature has attributed this variability to the task (Saucedo-Marquez et al., 2013; Karok et al., 2017), duration of stimulation (Puri et al., 2016), or electrode montage (Tazoe et al., 2014). Previous works argue that these fairly contradictory results may be explained by individual differences among study participants (Bikson et al., 2012; Li et al., 2015), as the effects of tDCS appear to be brain state-dependent (Bikson et al., 2016). Thus, it seems to be critically determined by the previous psychological state of the stimulated participant, including baseline gamma-aminobutyric acid (GABA) levels, individual circadian rhythms, genetics, brain injury and the initial state of the motor and cognitive function (Li et al., 2015), which configured the focus of the present work.

Musicians are considered a human model of inter-individual differences for studying behavioral-cognitive processes and brain effects of acquiring, practicing, and maintaining specialized motor skills (Schlaug, 2015). Musical training seems to shape certain brain areas through neuroplasticity mechanisms, as neuroimaging techniques have demonstrated differences in structures and functions of the motor regions of musically trained individuals, especially those areas related to auditory and sensorimotor networks (Gaser and Schlaug, 2003a,b; Bengtsson et al., 2005; Bangert et al., 2006; Baumann et al., 2007; Hyde et al., 2009; Herholz and Zatorre, 2012; Steele et al., 2013; Zamorano et al., 2017), leading to a better motor performance (Scheurich et al., 2018). These differences lead to an enhanced motor function. For instance, when tapping a specific rhythm, musicians have shown a more synchronized and flexible tapping rate, as well an enhanced error correction mechanism than non-musicians (Scheurich et al., 2018). In addition, musicians seem to learn faster during a motor sequence tapping task compared to a control group (Tucker et al., 2016) and have showed to be more precise than controls in a circle-drawing task (Janzen et al., 2014). Moreover, Spilka et al. (2010) showed that musicians were able to imitate hand movements during video clip watching more accurately than non-musicians (Spilka et al., 2010). Therefore, Gorniak et al. (2018) proposed that musicians are a unique population with respect to fine motor control of the hand that show cortical reorganization, and suggest that this population should be studied separately from typical healthy controls with respect to hand function (Gorniak et al., 2018).

The Sequential Finger Tapping task (SEQTAP) is one of the most used tasks to measure motor tapping in healthy subjects. The application of anodal tDCS over M1 during three consecutive days has previously been shown to reduce significantly the reaction time in the finger tapping task/serial reaction time task (SEQTAP/SRTT) (Hashemirad et al., 2016). Evidence supports the hypothesis that timely co-application of (hand/arm) training and tDCS to the contralateral M1 facilitates long-term memory formation, reflecting use-dependent plasticity (Rroji et al., 2015). Nevertheless, data on the effects of tDCS on the retention of the skill in the SEQTAP remain controversial. Saucedo-Marquez et al. (2013) found that intervention with tDCS on M1 during three consecutive days improved the performance in the SEQTAP task during the stimulation, but the effect of tDCS was diminished 1 week after the stimulation. Accordingly, Schambra et al. (2011) found offline effects of tDCS over the Sequential Visual Isometric Pinch Task (SVIPT), but with a low effect size. A recent meta-analysis concluded that the effects of a-tDCS over the non-dominant M1 generally diminish after 24 h (Dissanayaka et al., 2017). Therefore, experimental studies are needed to define the stimulation protocols that produce the retention of the skill over sequence motor learning.

Hence, the aim of the present work was to evaluate whether previous musical training differentially impacts the effect of tDCS, and to evaluate the effects of tDCS on a motor sequence-learning task during and after stimulation. For this purpose, we applied a-tDCS over the right M1 during three consecutive sessions of performance of the SEQTAP and registered the skill index (SI) exhibited during the stimulation of musicians and non-musicians. In addition, we performed two follow-up measurements 20 min and 8 days after the stimulation in order to assess the effect of tDCS on the retention of the skill.

## Materials and Methods

### Participants

Forty six healthy subjects participated in the study. The participants were all undergraduate students from the University of Almeria. Inclusion criteria were as follows: (1) right handedness; (2) no metallic implants on the head area; (3) no recent consumption of drugs or psychotropic medication; (4) no diagnosed psychopathology per the Diagnostic and statistical manual of mental disorders (5th Edition) (DSM-5); (5) no history of epilepsy, (6) naivety to the task and to tDCS, (7) and to score in the SEQTAP task between the 2nd and the 98th percentile. Participation was voluntary and academically rewarded. Two types of participants were recruited: musicians (M) and non-musicians (nM). Inclusion criteria for musicians were: (1) playing of a musical instrument at least once per week during a year; (2) the played instrument required the left hand; and (3) the instrument was practiced within the last 3 months. We counted in total with *n* = 19 musicians: seven piano players (5.14 ± 3.48 years of experience), nine guitar players (4.44 ± 4.18 years of experience), two drums players (4.00 ± 2.82 years of experience) and four saxophone players (10.50 ± 1.73 years of experience). Three out of 19 participants played two instruments.

Participants were randomly assigned to the tDCS or sham condition using Microsoft Excel software. After statistical analysis, four participants were detected as outliers: two participants scored above the 98th percentile and two participants scored below the 2nd percentile in one of the SEQTAP tests. Two participants presented missing data in at least one test. Therefore, the present study included 40 participants with an age range of 18–32 years (mean = 20.77 years, *SD* = 3.50), of which 70% were female, distributed into four groups: musicians-tDCS (M-tDCS) (*n* = 9) (19.36 ± 1.75 years old, 6 female), musicians-sham (M-sham) (*n* = 10) (21.00 ± 3.26 years old, 5 female), non-musicians-tDCS (nM-tDCS) (*n* = 11) (22.10 ± 4.17 years old, 7 female) and non-musicians-sham (nM-sham) (*n* = 10) (20.77 ± 4.41 years old, 9 female).

Volunteers gave their informed consent to participate in the study, which was undertaken in accordance with the ethical standards of the World Medical Assembly (WMA) Declaration of Helsinki on the Ethical Principles for Medical Research Involving Humans. All personal information was handled under the Spanish personal data protection law of the 13th December 15/1999. The experimental procedure was approved by the Committee on Bioethics in Human Research (CIH) of the University of Almeria, Spain.

### Sequential Finger Tapping Task (SEQTAP)

The SEQTAP task is a commonly used motor sequence learning task in which the participant learns to type, as quickly and accurately as possible, a sequence of numbers with the non-dominant hand. Participants respond to a series of five digits, ranging from 1 to 4 on a computer screen by pressing the corresponding button with the corresponding finger on a keyboard (Walker et al., 2002). We adapted the task from Saucedo-Marquez et al. (2013) with the following modifications: the time of each block was reduced from 40 to 20 s and the inter-block time was reduced from 20 to 10 s. Better performance in keyboard training has been found when training trials are distributed over time rather than blocked together (Baddeley and Longman, 1978). In addition, we attempted to reduce the fatigue of the participants by using a more distributed learning method. This structure permitted the participants to complete ˜2 blocks per minute over 20 min. The task was completed with the left (non-dominant) hand and was programmed with E-Prime Professional v. 2.0.8.74.

The measure registered during the SEQTAP task was the SI, obtained by dividing the percentage of correct sequences by the average time per trial (Cuypers et al., 2013):

$$SI=\frac{\%\;Correct\;Sequences}{mean\;respone\;time\;per\;20s\;trial}$$

### Transcranial Direct Current Stimulation (tDCS)

Transcranial direct current stimulation was administered with a Magstim DC-Stimulator Plus from neuroConn (Ilmenau, Germany) on the right M1 according to the 10–20 mm international EEG system. The selected area and both electrodes were soaked in physiological saline (∼20 ml per session). The excess of saline was eliminated with a clean dry towel. The anode (5 cm × 4 cm) was placed in the selected area (C4) by a tDCS cap and the cathode (5 cm × 4 cm) was placed in the contralateral trapeze and attached with hypoallergenic adhesive tape. Unicephalic stimulation was used, as outcome measures in motor sequence learning have reported no differences between unicephalic and bicephalic tDCS (Hashemirad et al., 2016). Moreover, the activity in the brainstem autonomic centers has not been shown to be modulated by the extracephalic location of the reference electrode (Vandermeeren et al., 2010). Stimulation was delivered at 2 mA (Iyer et al., 2005; Galea and Celnik, 2009), and thus applied with a current density of 0.10 mA/cm^2^, a level considered to be within the safe parameters (Iyer et al., 2005) which has previously shown to improve online performance gains and the offline maintenance for implicit motor sequence learning (Kantak et al., 2012; Sánchez-Kuhn et al., 2017). Stimulation was delivered during the complementation of the 3 SEQTAP sessions over 20 min (fade-in and fade-out of 30 s). In the sham condition, the stimulation lasted only for the first minute plus a fade-in and fade-out of 30 s. Discomfort was monitored using verbal open-ended questions.

### Experimental Procedure

The experiment was conducted in an artificially lit room held at approximately 22°C on a computer not connected to electricity for the duration of the experiment.

A previous pilot experiment was carried out on four participants: two males and two females. Possible side effects or causes of discomfort were noted. Mild itching was noted in one of four participants in the area of the electrode and seemed to be dependent on the volume of saline used. Therefore, the volume of saline for the sponge was adjusted to 20 ml per session, following Raw et al. (2016), as a higher volume of saline, but not oversaturated sponges, appear to be related to less discomfort (Woods et al., 2016). Hence, the excess of saline was dried to avoid dripping.

**FIGURE 1:**
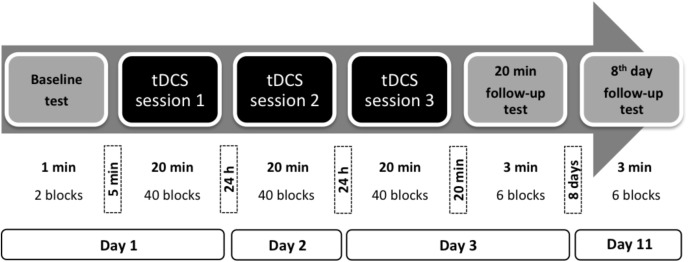
Experimental procedure.

As depicted in **Figure 1**, each participant received tDCS or sham for three consecutive days during the performance of the SEQTAP in sessions 20 min in duration. The baseline test of the SEQTAP (1 min duration) was performed 5 min prior to session 1. Following session 3, follow-up tests were conducted 20 min and 8 days later (8th day follow-up test), each 3 min in duration. The experiment was conducted on afternoons between Monday and Thursday over 16 weeks.

### Statistical Analysis

In instances were data was normally distributed (Shapiro–Wilk test: *p* ≥ 0.05), we tested skewness and kurtosis to confirm a uniform distribution, and statistical differences were calculated by the Analysis of Variance (ANOVA) with Bonferroni *post hoc*. Effect size (η_p_^2^) was calculated for all the significant results and interpreted following Cohen’s classification: (0.1 – small size, 0.3 – medium size, and 0.5 – large size) (Cohen, 1973). If data was non-normally distributed, statistical differences were estimated using a Kruskal–Wallis and U Mann–Whitney test. Effect size (r) for U Mann-Whitney results was calculated for all the significant results, with the previously mentioned classification. Statistical significance was set up at *p* ≤ 0.05. Possible statistical differences among age and gender were assessed using Pearson’s correlation (2-tailed). Analyses were executed using SPSS Version 24.0 software (IBM Corp, Armonk, NY, United States).

## Results

The obtained results are displayed in **Table 1**.

**Table 1:** Shows the Mean (M) and Standard Deviation (SD) Skill Index scores (SI scores) obtained by each group in each test.

	M-tDCS		M-sham		nM-tDCS		nM-sham	
Test	*M*	*SD*	*M*	*SD*	*M*	*SD*	*M*	*SD*
BL	0.029	0.010	0.024	0.013	0.017	0.011	0.014	0.006
S1	0.058	0.013	0.055	0.025	0.044^∗^	0.012^∗^	0.034^∗^	0.007^∗^
S2	0.076	0.018	0.076	0.035	0.058^∗^	0.010^∗^	0.045^∗^	0.016^∗^
S3	0.086	0.021	0.081	0.037	0.064	0.012	0.053	0.020
20 min	0.097	0.026	0.084	0.047	0.072^∗^	0.013^∗^	0.058^∗^	0.023^∗^
8th day	0.101	0.024	0.081	0.036	0.072^∗^	0.014^∗^	0.053^∗^	0.018^∗^

*M-tDCS, Musicians tDCS; M-sham, Musicians sham; nM-tDCS, Non-musicians tDCS; nM-sham, non-musicians sham; BL, baseline test; S1, tDCS session 1; S2, tDCS session 2; S3, tDCS session 3; 20 min, 20 min follow-up test; 8th day, 8th day follow-up test; ^∗^p < 0.05*.

**FIGURE 2:**
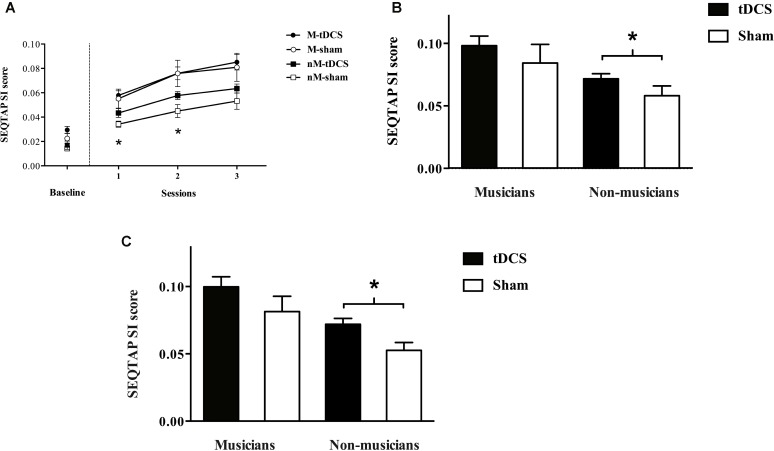
**(A)** Shows the mean ± SEM SI scores obtained by each of the four groups across the baseline test and the three sessions of tDCS/sham (S1, Session 1; S2, Session 2; S3, Session 3 (^∗^*p* ≤ 0.05). **(B)** Shows the mean ± SEM SI scores obtained by each of the four groups across in the 20-min follow-up test (**^∗^***p* ≤ 0.05). **(C)** Shows the mean ± SEM SI scores obtained by each of the four groups in the 8th day follow-up test (**^∗^***p* ≤ 0.05).

In the baseline test (**Figure 2A**), results showed significant differences in the SI score between musicians and non-musicians (0.023 ± 0.010 and 0.016 ± 0.010, respectively) [*F* (1, 38) = 11.755, *p* = 0.001; η_p_^2^ = 0.24]. No previous differences were found in the baseline test between the nM-tDCS group and the nM-sham group (*U* = 36, *p* = 0.49), nor between the M-tDCS group and the M-sham group (*U* = 29, *p* = 0.07).

Within the three sessions of tDCS results showed a group effect in session 1 [χ^2^(3) = 16.205, *p* = 0.001], session 2 [χ^2^(3) = 15.799, *p* = 0.001], and in session 3 [χ^2^(3) = 12.911, *p* = 0.001]. Specifically, in the non-musicians, analysis revealed statistically significant differences between the nM-tDCS group and the nM-sham group in session 1 (*U* = 16; *p* = 0.01; *r* = 0.42) and session 2 (*U* = 18; *p* = 0.02; *r* = 0.43). No statistically significant differences were seen in session 3 (*U* = 27, *p* = 0.15).

In the musicians, no statistically significant differences were found between the M-tDCS and the M-sham group in session 1 (*U* = 53, *p* = 0.91), session 2 (*U* = 52, *p* = 0.86), or session 3 (*U* = 52, *p* = 0.86). At the 20-min follow-up test (**Figure 2B**), there was a group effect [*F*(3, 36) = 4.839, *p* = 0.001], and *post hoc* analysis revealed significant differences between the nM-tDCS group and the nM-sham (*p* = 0.03; *η_p_^2^* = 0.20), but not between the M-tDCS group and the M-sham group (*p* = 1.00).

At the 8th day follow-up test (**Figure 2C**), there was a group effect [χ^2^(3) = 15.799, *p* = 0.001]. Specifically, there were statistically significant differences between the nM-tDCS group and the nM-sham group (*U* = 18, *p* = 0.02; *r* = 0.88). However, there were no significant differences between the M-tDCS group and the M-sham group (*U* = 52, *p* = 0.863).

There was a significant negative correlation between age and the SI scores of the baseline test (*r* = –0.494; *n* = 40; *p* = 0.001). No other significant correlation was found between age or gender and the SI scores of the baseline test, the tDCS sessions, the 20-min follow-up or the 8th day follow-up test. Five of 40 participants reported fatigue due to performance of the task in at least one test. The perceived sensations during the tDCS stimulation are reported in **Table 2**.

**Table 2:** Shows the number of participants of each group that reported sensations during the stimulation.

Sensation	Anode group (*n* = 20)	Sham group (*n* = 20)
	Number of	Number of
	participants	participants
Itch	16	16
Heat	1	1
No sensation	3	3

*Itch, Mild itching at the beginning of the stimulation (*˜*1 min); Heat, Heat feeling in the electrode placement.*

## Discussion

The aim of the present study was to explore the effect of three sessions of tDCS over the motor cortex of musicians and non-musicians during the performance of a motor learning task. Performance was also assessed in follow-up tests of 20 min and 8 days.

In the non-musicians, we found an effect of tDCS at the first and second session of stimulation, in the 20 min and in the 8th day follow-up test. In the musicians, we found no effect of tDCS in any of the tests. Therefore, results show that tDCS may help in the learning and maintenance of newly acquired motor abilities but that the effect is dependent on previous musical training.

Musicians showed better performance in the SEQTAP compared to non-musicians in the baseline test. It might be possible that previous musical training shapes brain structures (Gaser and Schlaug, 2003a; Hyde et al., 2009), the myelinisation (Bengtsson et al., 2005; Steele et al., 2013) and consequently the behavioral outcomes (Xie et al., 2013) due to plasticity processes. Therefore, the present results confirm the condition of previous musical training as a suitable human model for studying individual differences among motor processes in healthy subjects (Schlaug, 2015).

During the three sessions of SEQTAP in which tDCS was administered, the principal effect of the stimulation was found in the nM group, as the nM-tDCS group scored significantly higher than the nM-sham group. This effect was seen in the first and in the second session, pointing toward a higher effect of tDCS at the beginning of the learning process. Ehsani et al. (2016) found similar effects of tDCS applied over M1, where the principal effect of tDCS was seen over the first blocks of a motor training task during a single stimulation session. Accordingly, Antal et al. (2004) found positive effects of tDCS on M1 in the early learning phase of a visuo-motor task. The present results support studies in which anodal stimulation strengthens newly formed associations (Orban de Xivry and Shadmehr, 2014), which seem to be primarily influenced by changes in membrane potential and GABAergic neurotransmission via interneurons of the neuronal network existing at a given time point (Nitsche et al., 2004, 2008). Previous studies have also suggested that the effects of tDCS are reduced as participants gain expertise, since participants may utilize different brain networks after the acquisition of the new task than before (Bullard et al., 2011; Clark et al., 2012). Thus, in order to optimize effectiveness in training progress, future studies should apply tDCS over different brain locations at different times during training.

The nM-tDCS group showed benefit from tDCS at the 20 min follow-up test and the 8th day follow-up. The results are in accordance with previous reports suggesting a role of M1 in the retention of newly acquired motor memories (Muellbacher et al., 2002; Hadipour-Niktarash et al., 2007; Galea and Celnik, 2009; Hunter et al., 2009). According to this assumption, the application of multiple sessions of tDCS over M1, compared to a single session tDCS, has been shown to induce significant changes in behavioral outcomes in the SEQTAP, particularly post-intervention (Hashemirad et al., 2016). This consolidation of newly acquired motor abilities in healthy participants over time is considered to be dependent on alterations in membrane potential and synaptic plasticity, specifically in glutamate and GABA signaling (Stagg and Nitsche, 2011). In contrast, Saucedo-Marquez et al. (2013) found no effects of tDCS in the SEQTAP retention test. The most notable difference between that study and the present work was the change in the length of the blocks in the SEQTAP, which were reduced from 40 to 20 s of tapping and 20 to 10 s of resting. These modifications could have reduced the fatigue of the participants, as only five of 40 participants reported fatigue in one or more sessions. Moreover, previous studies have shown better performance in keyboard training when training trials were distributed over time rather than grouped together (Baddeley and Longman, 1978), as spaced learning protocols might yield to better outcomes than massed ones (Smolen et al., 2016).

The fact that tDCS did not produce any differences between the stimulated and the sham group in the musicians supports the idea that inter-individual factors can vary the responses to tDCS (Li et al., 2015). One of the factors that could predict the effect of tDCS might be the baseline performance. The initial motor function state of the participant can have a meaningful impact on the effects of tDCS, as previous studies have shown that participants with poorer selective muscle activation improved more after the stimulation of tDCS on M1 (Uehara et al., 2015). In addition, Ciechanski et al. (2017) found a greater effect of tDCS over neurosurgical skill acquisition in low, rather than in high-skill trainees. These findings can be extended also to other study fields, such as lateralised visual detection task sensitivity. For instance, in a study carried out by Learmonth et al., participants were divided into “poor performers” (lower d’) and “good performers” (higher d’). tDCS was applied to the left posterior parietal cortex (PPC) and only poor performers got benefitted from the stimulation (Learmonth et al., 2015). A further study involving musicians found that novice jazz players’ musical performance was enhanced by tDCS applied over the right dorsolateral prefrontal cortex (r-DLPFC), while experienced jazz players’ musical performance was unchanged and even deteriorated following stimulation (Rosen et al., 2016). A High-Definition tDCS (HD-tDCS) study also reported a baseline-dependent effect of stimulation, showing greater benefit for those participants with poorer baseline scores (Shen et al., 2016). The fact that participants with a higher level of baseline performance experience less benefit from tDCS should be taken into account in future tDCS studies. Future works should include other populations with high motor capacities such as gamers, who have been shown to have signs of brain reorganization due to motor expertise similar to professional musicians (Granek et al., 2010).

The ineffectiveness of tDCS over motor capacity in musicians has also been reported by Furuya et al. (2013), who found no apparent improvement in the fine control of finger movements in professional pianists, while untrained individuals benefited from tDCS. This finding has two possible explanations. One reason for this unsuccessful effect of tDCS has been attributed by previous studies to a ceiling effect displayed by high-performing musicians (Furuya et al., 2014). This explanation is not plausible for the present study, as the musician group show an improved performance over the three sessions. However, neuroimaging studies offer a second possible explanation for this finding: functional magnetic resonance imaging (fMRI) in professional piano players during complex finger movement task training showed significantly lower activation clusters in M1, supplementary motor area, premotor cortex, and superior parietal lobule when compared to a control group (Krings et al., 2000). Therefore, it might be possible that during motor learning, professional musicians display a reduced level of brain activity in areas required for the control of basic movement (Koeneke et al., 2004; Granek et al., 2010; Wright et al., 2012). In addition, neuroimaging studies have pointed toward a larger use of other brain areas by musicians during learning and memorization, such as the superior parietal cortex, the supramarginal gyrus, and the cerebellum (Lotze et al., 2003; Stewart et al., 2003). These brain areas might be addressed in future clinical studies of tDCS involving musicians.

The limitations of the study are mostly related to the sample size and sample heterogeneity. However, in the present study, each participant was requested to assist four different days to the laboratory, which means a high time cost for the participant and made difficult the recruitment of the sample. One of the most remarkable limitations is the predominance of women in the composition of the subgroups. Although no differences were found in gender in the present study, future tDCS studies should address an equal gender distribution, as tDCS has shown to be sensible to individual differences such as this (Chew et al., 2015). Aiming at obtain a higher size effect, the present study needs further replication counting with a larger sample.

## Conclusion

In conclusion, the present study showed that tDCS applied over the right M1 had a positive effect on motor learning in healthy non-musician participants, enhancing motor performance at the first and second session of tDCS, showing a maintenance of this effect 20 min and 8 days after the intervention. Therefore, the current experiment offers a protocol that allows the study of both online and offline effects of tDCS among healthy participants. These results enhance tDCS as a complementary technique for motor neurorehabilitation, particularly for its long-term potentiation effects. However, the beneficial effects of tDCS were not observable in musically trained participants. Consequently, it is plausible to conclude that tDCS on M1 has different effects depending on previous motor experiences, which highlights the importance of individual differences when considering the effects of tDCS.

## Author Contributions

AS-K performed the collection, analysis, and interpretation of the data and drafted the paper. CP-F and MM reviewed the paper and provided critical intellectual output to the work. FS-S and PF performed the main conception and design of the work, interpretation of data, and reviewed and commented the paper. All authors read and approved the final version to be published.

## Conflict of Interest Statement

The authors declare that the research was conducted in the absence of any commercial or financial relationships that could be construed as a potential conflict of interest. The reviewer RR andhandling Editor declared their shared affiliation at the time of the review.
